# Extended duration of treatment using reduced-frequency dosing of anti-PD-1 therapy in patients with advanced melanoma and Merkel cell carcinoma

**DOI:** 10.1007/s00262-023-03539-8

**Published:** 2023-09-21

**Authors:** Lisa May Ling Tachiki, Daniel S. Hippe, Karly Williams Silva, Evan Thomas Hall, William McCamy, Dane Fritzsche, Andrea Perdue, Julia Majovski, Thomas Pulliam, Daniel A. Goldstein, Joshua Veatch, Joel Ho, Paul T. Nghiem, John A. Thompson, Shailender Bhatia

**Affiliations:** 1 Department of Medicine, Division of Medical Oncology, University of Washington, Seattle, WA USA; 2 Department of Medicine, Division of Dermatology, University of Washington, Seattle, WA USA; 3 Clinical Research Division, Fred Hutchinson Cancer Center, Seattle, WA USA; 4 Davidoff Cancer Center, Rabin Medical Center, Petah Tikva, Israel

**Keywords:** Immunotherapy, Nivolumab, Pembrolizumab, Melanoma, Merkel cell carcinoma, Drug costs

## Abstract

**Background:**

Optimal duration of treatment (DoT) with immune checkpoint inhibitors (ICI) in metastatic cancers remains unclear. Many patients, especially those without radiologic complete remission, develop progressive disease after ICI discontinuation. Extending DoT with ICI may potentially improve efficacy outcomes but presents major logistical and cost challenges with standard frequency dosing (SFD). Receptor occupancy data supports reduced frequency dosing (RFD) of anti-PD-1 antibodies, which may represent a more practical and economically viable option to extend DoT.

**Methods:**

We conducted a retrospective study of patients with metastatic melanoma and Merkel cell carcinoma (MCC), who received ICI at RFD administered every 3 months, after initial disease control at SFD. We evaluated efficacy, safety, and cost-savings of the RFD approach in this cohort.

**Results:**

Between 2014 and 2021, 23 patients with advanced melanoma (N = 18) or MCC (N = 5) received anti-PD-1 therapy at RFD. Median DoT was 1.1 years at SFD and 1.2 years at RFD. The 3 year PFS after start of RFD was 73% in melanoma and 100% in MCC patients, which compare favorably to historical control rates. In the subset of 15 patients who received at least 2 years of therapy, total savings amounted to $1.1 million in drug costs and 384 h saved despite the extended DoT (median 3.4 years), as compared to the calculated cost of 2 years at SFD.

**Conclusions:**

ICI administration at RFD can allow extension of treatment duration, while preserving efficacy and reducing logistical and financial burden. RFD approach deserves further exploration in prospective clinical trials.

## Introduction

Immune checkpoint inhibitors (ICI) have led to remarkable improvement in the patient outcomes in several different cancer types, including metastatic melanoma and Merkel cell carcinoma (MCC) [1–4]. Although a sizable proportion of patients with these aggressive skin cancers experience initial responses and durable clinical benefit with ICIs, the optimal duration of treatment to sustain long-term disease control remains unclear [5, 6]. Currently, the clinical approaches for patients who are benefitting from ICI include continuation of standard doses of ICI therapy until disease progression or discontinuation of therapy after 2 years, as per the KEYNOTE-006 protocol [1]. Durable complete responses (CR) after treatment discontinuation have been observed in a select cohort of metastatic melanoma patients treated in KEYNOTE-001 [7]. However, ICI discontinuation in melanoma patients without a CR and in MCC patients may be associated with a higher rate of progression over time, as compared to historical outcomes with ICI continuation [8–11]. For these patients, continuing immunotherapy may be beneficial to sustain treatment outcomes. Yet, indefinite continuation at standard frequency doses (SFD) poses substantial logistical and cost drawbacks [12, 13].

Rather than discontinuing ICI therapy at an arbitrary time point, optimizing dose frequency may be an alternative way to decrease the use of this expensive class of drugs while maintaining anti-tumor responses [6]. In phase I studies, a single dose of nivolumab was associated with sustained PD-1 receptor occupancy on peripheral blood T cells up to 100 days [14]. This pharmacodynamic data suggests that clinical benefit from ICI therapy could be achieved with less frequent doses of anti-PD-1 agents [15]. Based on this data, we implemented a reduced frequency dosing of anti-PD-1 agents administered every 3 months to mirror the observed duration of PD-1 receptor occupancy in phase 1 studies. We have employed reduced frequency dosing (RFD) of anti-PD-1 antibodies as an alternative approach to extending the duration of treatment in our clinic patients, while mitigating the financial and logistical burdens of ICI therapy. We discuss this approach of de-escalating treatment with RFD with our patients generally after they appear to have experienced maximal response to standard ICI therapy. The decision to transition to RFD is influenced by the timing and depth of disease response for each patient, rather than an arbitrary duration on SFD.

Reported clinical outcomes from extended-interval ICI dosing are limited [16]. The purpose of this study was to retrospectively evaluate the efficacy and safety of RFD of anti-PD-1 antibodies at our institution in patients with advanced melanoma and Merkel cell carcinoma. To our knowledge, this is the first study to report efficacy outcomes of RFD in patients with melanoma and MCC and to provide a cost-savings analysis from implementing RFD.

## Methods

We conducted a single-center, retrospective analysis of patients with advanced melanoma or MCC treated with anti-PD-1 or anti-PD-L1 therapy at the Fred Hutchinson Cancer Center between January 2014 and June 2021. This study was approved by the local Institutional Review Board and was conducted in accordance with the Declaration of Helsinki provisions.

Eligible patients were at least 18 years of age and had a confirmed diagnosis of either melanoma (excluding mucosal and uveal melanoma) or MCC with distant metastatic or unresectable locoregional disease. All patients had received anti-PD-1/PD-L1 therapy administered initially at standard frequency dosing (SFD) but had subsequently transitioned to reduced frequency dosing (RFD) after discussions with the treating clinicians. Transition from SFD to RFD was a shared clinical decision made by the patients after a thorough, documented discussion with their treating clinician. The decision was influenced by the timing and depth of disease response, logistical demands of SFD, toxicity considerations and importantly, patient preference and comfort around the potential risks of a non-standard dosing schedule. Many patients opted for a stepwise de-escalation from standard frequency doses to every 2 month doses initially. Once progression-free status was maintained on every 2 month doses, patients felt more comfortable with transitioning to every 3 months doses of anti-PD-1 therapy. Hence, the time to transition was variable across this cohort of patients.

SFD was defined as any of the prior and current FDA-approved administration regimens for pembrolizumab (2 mg/kg or 200 mg every 3 weeks; 400 mg every 6 weeks), nivolumab (3 mg/kg or 240 mg every 2 weeks; 480 mg every 4 weeks) or avelumab (10 mg/kg or 800 mg every 2 weeks), either as monotherapy or in combination with other immunotherapy agents such as ipilimumab [17, 18]. RFD was defined as pembrolizumab or nivolumab administered at a frequency less than SFD, once every 8–12 weeks.

Patient demographics, location of metastases at initiation of anti-PD-(L)1 therapy, prior lines of treatment, dates of immunotherapy treatment, immune-related adverse events (irAE), time to progression, and date of last follow-up were collected from patient medical records. Efficacy assessments included best overall response (BOR) on SFD, progression free survival (PFS), and overall survival (OS). BOR was defined as the best response category, per RECIST 1.1, recorded from the start of anti-PD-1 therapy at SFD prior to the transition to RFD. All patients were experiencing clinical benefit with SFD prior to the transition. Eligible patients were also required to have obtained ≥ 1 radiographic scan after transitioning to RFD to evaluate for disease response.

PFS was calculated as the time interval from the date of the first infusion at RFD to the earliest subsequent date of progression or death. Patients who were alive without progression were censored at last follow up. Similarly, time to irAE was calculated starting from the date of the first infusion at RFD and censored at last follow up. Progression and death were considered competing risks for irAEs. Rates of irAE were summarized for all irAEs (any grade) and for grade 3/4 irAEs. PFS was estimated using Kaplan–Meier methods, and rates of irAE were estimated using the cumulative incidence function estimator to account for competing risks. Confidence intervals (CIs) for PFS and irAE rates were calculated using conventional standard error formulas except when the estimated rates were 0% or 100%. In those cases, CIs were calculated using the Clopper-Pearson exact method. All statistical calculations were conducted with the statistical computing language R (version 4.0.3; R Foundation for Statistical Computing, Vienna, Austria).

### Economic analysis

Overall costs for the entire cohort were evaluated. The costs of RFD were calculated based on summative costs of administered doses of anti-PD-1 therapy for each patient. In patients who met or exceeded 2 years of therapy, the costs of RFD at extended duration of therapy were compared to the cost of 2 years of ICI therapy at SFD followed by treatment discontinuation. In patients who did not complete up to 2 years of treatment at the data cutoff date, the costs incurred by patients on RFD were compared to the costs needed to maintain the equivalent duration of ICI treatment at SFD. We used the average sales price (ASP) from the Center for Medicare and Medicaid Services for Part B drugs from the first quarter of 2021: $28.90 USD per mg for nivolumab to $51.35 USD per mg for pembrolizumab. We also collected estimated patient travel expenses by recording distances traveled between a patient’s home address and the cancer clinic. We multiplied the miles traveled by the U.S. Internal Revenue Service travel reimbursement rate for 2021: $0.56/mile. To calculate total patient time spent in outpatient clinic visits, we used published estimates for average patient time associated with travel (32 min), phlebotomy (51 min), physician interaction time (29 min), provider wait times (35 min), infusion wait times (58 min), and anti-PD-1 administration (30 min) [17–21].

## Results

### Patient characteristics

Of 257 patients with melanoma and MCC who were treated with anti-PD-(L) 1 therapy from January 2014 to June 2021, 23 patients with either metastatic melanoma (N = 18) or MCC (N = 5) received anti-PD-1 therapy at RFD. The characteristics of our retrospective cohort are displayed in Table 1. The median age of the patients was 61 years (range 40–92). Eighteen (78%) patients were male. Nine (39%) patients had previously received systemic therapy for advanced disease. At the time of starting anti-PD-1 therapy, seven (30%) patients had CNS metastases, and seven (30%) patients had visceral metastases without CNS disease. Nine (39%) patients received nivolumab therapy, and 14 (61%) patients received pembrolizumab. None of the patients in our cohort received avelumab or other anti-PD-(L) 1 agents.

**Table 1:** Patient demographics and clinical characteristics

Characteristic	All patients (N = 23)
Age in years; median (range)	60.5 (40–92)
Male sex—no. (%)	18 (78%)
ECOG performance score—no. (%)	
0	18 (78%)
1	3 (13%)
2	2 (9%)
Disease type—no. (%)	
Cutaneous melanoma	18 (78%)
Merkel cell carcinoma	5 (22%)
Elevated baseline LDH level—no. (%)	6 (26%)
Sites of metastases at therapy initiation—no. (%)^a^	
CNS metastases	7 (30%)
Visceral metastases	7 (30%)
Lung metastases	3 (13%)
Skin/Lymph node only metastases	6 (26%)
Anti-PD-1 agent—no. (%)	
Nivolumab	9 (39%)
Pembrolizumab	14 (61%)
Immunotherapy regimen—no. (%)	
Monotherapy	16 (70%)
With ipilimumab (during induction)	7 (30%)
Line of therapy for anti-PD-1 mAb—no. (%)	
1	14 (61%)
2 or higher	9 (39%)
Best response at transition to RFD—no. (%)	
Complete response	6 (26%)
Partial response	11 (48%)
Stable disease	6 (26%)

*ECOG *Eastern Cooperative Oncology Group performance status, *CNS *Central nervous system, *mAb *Monoclonal antibody, *RFD *Reduced frequency dosing

^a^Patients were classified by their most advanced site of metastatic disease. Patients with CNS metastases included patients with or without skin, soft tissue, lung or visceral sites of disease. Patients with visceral metastases included patients without CNS disease but may include patients with or without skin, soft tissue, and lung disease. Patients with lung metastases included patients with or without skin and soft tissue disease

### Treatment outcomes

**Fig. 1:**
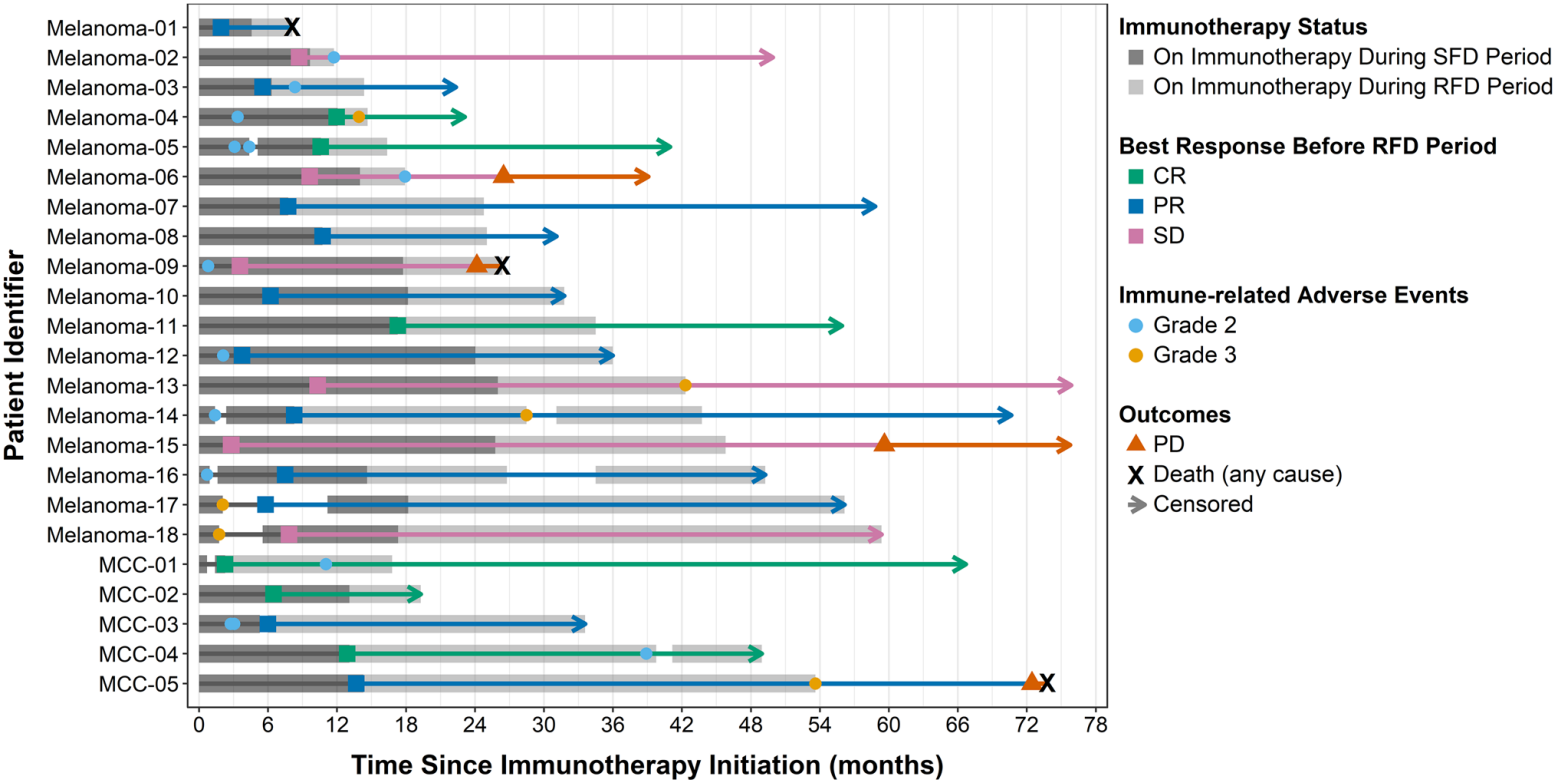
Clinical course of each patient receiving standard-frequency anti-PD-1 therapy followed by reduced-frequency dosing. This swimmer’s plot portrays duration of treatment, best overall response, clinically significant immune-related adverse events, and disease progression or death in 23 patients who received standard frequency dosing of anti-PD-1 (SFD) then transitioned to reduced-frequency dosing (RFD). Each patient received SFD (dark gray) until objective response or disease control was achieved, followed by transition to RFD (light gray) guided by timing of disease response. The median duration of therapy at SFD was 13.1 months (range 2.3–26.0), and the median duration of RFD was 14.5 months (range 2.1–42.1). *Abbreviations* SFD—Standard frequency dosing, RFD—Reduced frequency dosing, CR—Complete response, PR—Partial response, SD—Stable disease, PD—Progressive disease

**Fig. 2:**
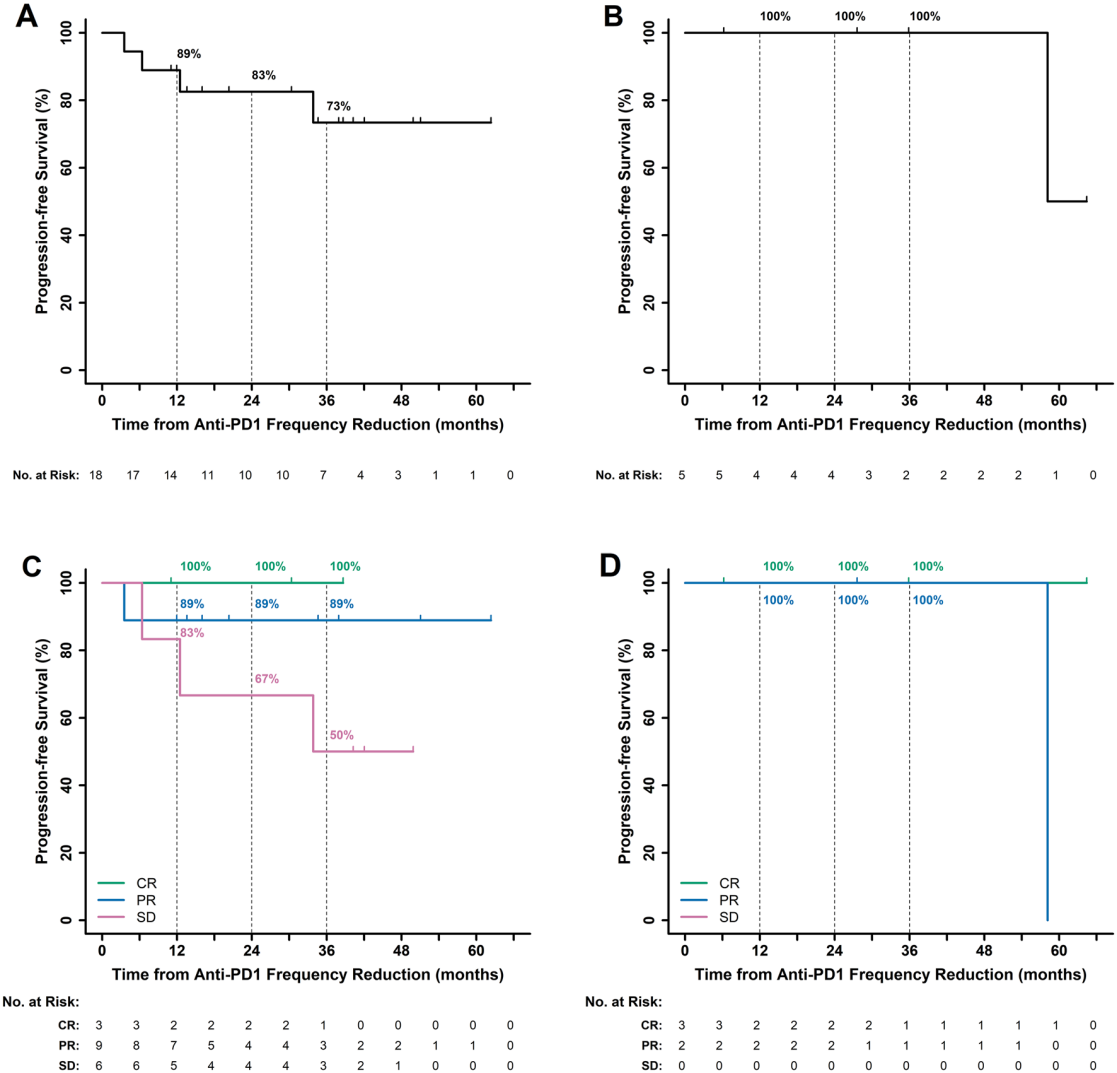
Kaplan–Meier curve showing progression-free survival (PFS) for patients by disease type and by best overall response. PFS was measured from initiation of reduced-frequency dosing to disease progression or death, whichever occurred first. Patients without an event were censored (tick mark) at the last disease assessment date. **A** From time of initiating reduced frequency dosing, the estimated 36 month PFS for melanoma patients was 73% (95% CI 53–100). Median PFS for melanoma patients was not reached. **B** The estimated 36 month PFS was 100% (95% CI 48–100). Median PFS for MCC patients was 58.2 months. **C** By best overall response (BOR) in melanoma, the estimated 36 month PFS rate for CR, PR, and SD were 100% (95% CI 29–100), 89% (95% CI 71–100) and 50% (95% CI 22–100), respectively. **D** By BOR in MCC, the estimated 36 month PFS rate was 100% (95% CI 29–100) in patients with CR and 100% (95% CI 16–100) in PR. *Abbreviations* PFS—Progression free survival, MCC—Merkel cell carcinoma, CR—Complete response, PR—Partial response, SD—Stable disease, PD—Progressive disease, BOR—Best overall response

Figure 1 demonstrates the clinical trajectory of each of the 23 patients, including the duration of treatment at SFD, the BOR achieved at SFD, duration of treatment at RFD, incidence of grade ≥ 2 irAEs, and disease progression or death, when applicable. At the time of data cutoff on July 8, 2021, the median follow-up duration from the start of ICI therapy at SFD was 49.0 months (range 8.1–75.9). From the first dose at SFD to the last dose at RFD, the median duration of total ICI therapy was 27.6 months (range 6.5–59.5). The median duration of therapy at SFD was 13.1 months (range 2.3–26.0). The BOR at SFD was CR (N = 6; 26%), PR (N = 11; 48%) or SD (N = 6; 26%). The median duration of therapy at RFD was 14.5 months (range 2.1–42.1). The 36 month PFS rate after the start of RFD was 73% (95% CI 53–100) in advanced melanoma (Fig. 2A) and 100% (95% CI 48–100) in advanced MCC (Fig. 2B). By BOR in melanoma, the 36 month PFS rate was 100% (95% CI 29–100) for CR, 89% (95% CI 71–100) for PR, and 50% (95% CI 22–100) for SD (Fig. 2C). In MCC, the 36 month PFS rate was 100% (95% CI 29–100) in patients with CR and 100% (95% CI 16–100) in PR (Fig. 2D). The median PFS after transition to RFD was not reached (95% CI not estimable) in melanoma patients and was 58.2 months in MCC patients (95% CI not estimable).

### Safety

During the SFD period, irAEs of any grade occurred in 48% of patients (N = 11), with grade 3 or higher events occurring in 9% of patients (N = 2). Two patients with irAE required systemic glucocorticoids to manage their toxicities, including grade 2 pneumonitis and grade 3 hepatitis. One patient required systemic glucocorticoids and infliximab to manage grade 3 colitis. The remaining eight patients were managed by withholding ICI therapy or adding hormone replacement. All patients had resolution of their irAEs prior to restarting immunotherapy. Upon reinitiating ICI therapy, only one of the 11 patients had recurrence of their prior irAE (neuropathy) during the RFD period.

**Fig. 3:**
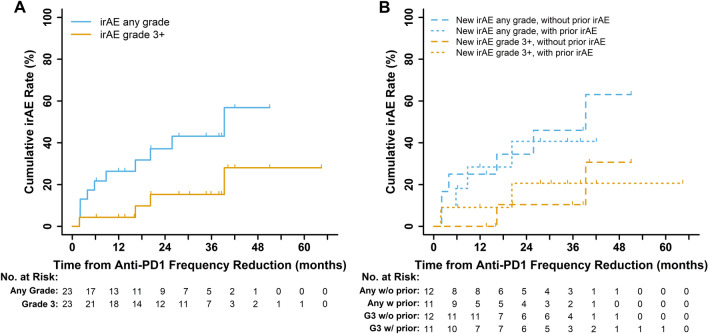
Cumulative rate of delayed immune-related adverse events (irAEs) in all patients on reduced frequency dosing (RFD). **A** The cumulative incidence of irAE after initiation of RFD was 57% (N = 10 patients) for all grades and 28% (N = 4 patients) for grade 3 irAEs. Grade 3 events included colitis (2), hepatitis, and dermatitis. **B** Rates of irAEs on RFD developed at similar rates between patients with a prior irAE during standard-frequency (N = 11) and patients who had never experienced an irAE previously (N = 12). Of the 11 patients who experienced irAE on standard-frequency doses of immunotherapy, only one patient experienced a recurrence of a prior irAE. Thus, the majority of delayed irAEs that patients incurred on RFD were new toxicity events. *Abbreviations* irAE—Immune-related adverse event, RFD—Reduced frequency dosing

There were 10 irAEs of any grade observed over the RFD period with a cumulative rate of 57% (95% CI 35–93). Eight of 10 events occurred within the first 24 months after starting RFD (1-year rate: 26%, 2 year rate: 37%). The last observed irAE occurred at 39 months after starting RFD (Fig. 3A). The cumulative rate of grade 3 irAEs was 28% (95% CI 11–72) during the RFD period. Four patients experienced a grade 3 irAE event, including colitis (N = 2), hepatitis (N = 1), and dermatitis (N = 1). Furthermore, irAEs on RFD occurred at similar rates between patients who had previously experienced an irAE during SFD and those without any prior irAE on SFD (Fig. 3B).

### Progression after RFD

**Fig. 4:**
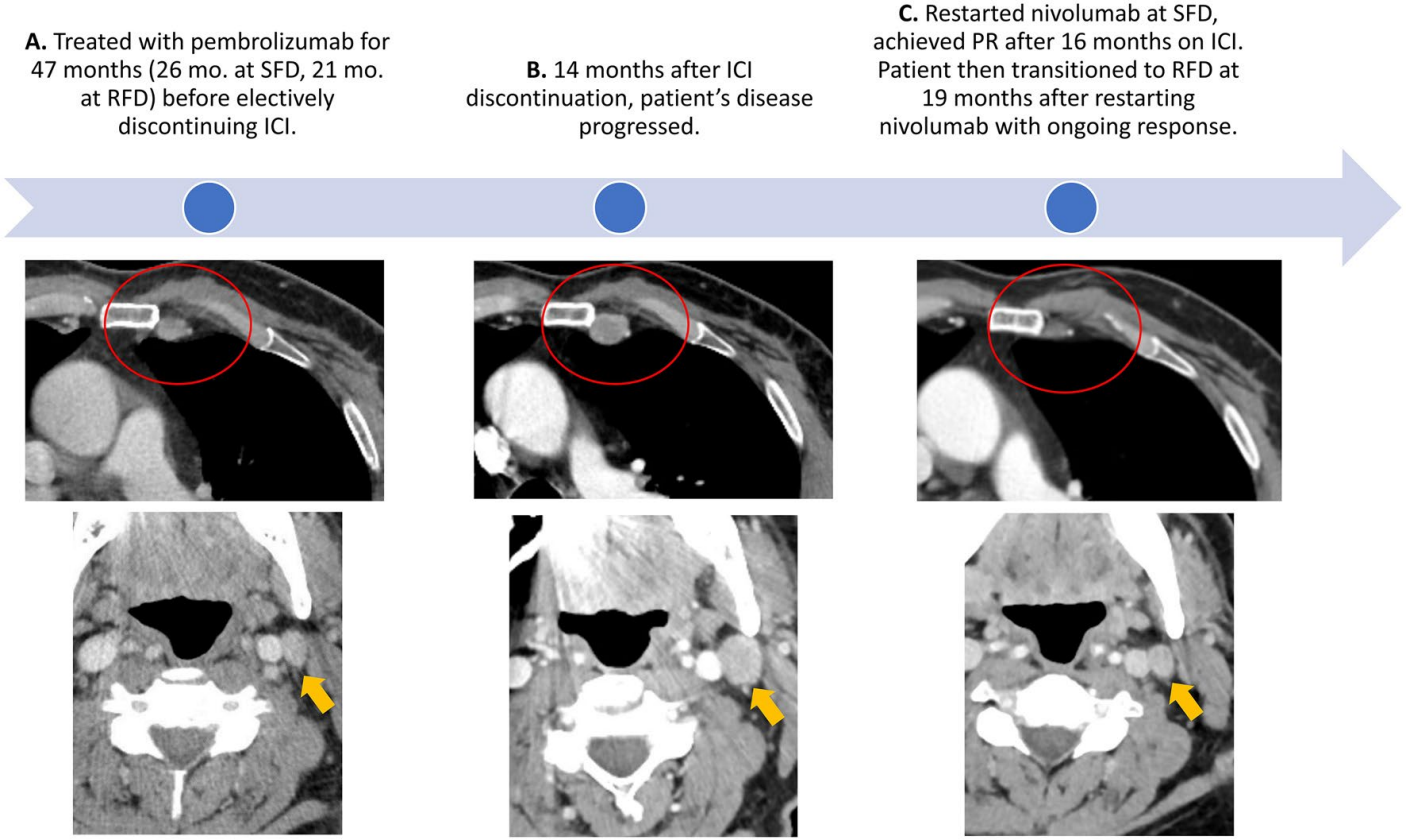
Case study suggesting dependence on continuous PD-1 blockade for disease control (patient Melanoma-15 from Fig. 1) Adult male with metastatic melanoma was treated with pembrolizumab for 47 months (26 months at standard-frequency dosing (SFD) and 21 months at reduced-frequency dosing (RFD) before he electively discontinued ICI (panel A). 14 months after elective discontinuation, his disease progressed (panel B), suggesting potential role of prolonged PD-1 blockade in controlling his disease. Reintroduction of nivolumab at SFD has recaptured his disease response (panel C), which is ongoing at 35 months after restarting nivolumab. This case suggests the importance of continuous PD-1 blockade in maintaining immune equilibrium in patients with residual disease

Four patients in our cohort had disease progression, summarized in Table 2. Only 1 patient developed progressive disease (PD), while still receiving treatment at RFD. This patient with metastatic melanoma had leptomeningeal involvement at baseline and had received nivolumab for 23 months (16 months at SFD plus 7 months at RFD) before developing recurrent melanoma in the leptomeninges. The other 3 patients had discontinued anti-PD-1 treatment (2 with forced discontinuation due to irAEs, and 1 with elective discontinuation) before developing PD several months (range 11–22 months) after treatment discontinuation. The patient with metastatic melanoma who electively discontinued anti-PD-1 therapy illustrates disease biology that may benefit from ongoing PD-1 blockade for long-term disease control. This patient had achieved SD as BOR while on pembrolizumab for a total duration of 47 months (26 months at SFD and 21 months at RFD), and then elected to discontinue therapy due to prolonged progression-free status (Fig. 4A). Unfortunately, 14 months after treatment discontinuation, he developed PD at the sites of prior metastases (Fig. 4B). He re-initiated anti-PD-1 therapy and experienced tumor regression suggesting disease sensitivity to PD-1 blockade (Fig. 4C). After 16 months on SFD, he was again transitioned to RFD with nivolumab and continues to experience disease control another 19 months later. His disease course suggests persistent residual melanoma cells that remain in prolonged immune equilibrium phase facilitated by ongoing PD-1 blockade. The RFD approach has majorly reduced the logistical burden of care during his prolonged disease course spanning more than 7 years.

**Table 2:** Outcomes of patients who developed disease progression during or after reduced-frequency dosing

	Disease type	Time on anti-PD-1 therapy	BOR 1st course	Status of anti-PD-1 therapy at progression	Site(s) of progression	New site of metastatic disease?	Therapy for PD	BOR 2nd course	Time on therapy	Disease status at follow up
1	Cutaneous melanoma	47 months (SFD 26; RFD 21)	SD	OFF therapy for 14 months electively	Lymph nodes	No	nivolumab (SFD 16 months; RFD 19 months)	PR	35 months	Ongoing partial response
2	Cutaneous melanoma	23 months (SFD 16; RFD 7)	SD	Receiving nivolumab 240 mg every 2 months	Leptomeninges	No	BRAF/MEK inhibitor therapy	PD	1.9 months	Died
3	Cutaneous melanoma	16 months (SFD 13; RFD 3)	SD	OFF therapy for 11 months due irAE (Hepatitis)	Brain	Yes	SRS; no systemic therapy	N/A	N/A	No evidence of disease progression for 36 months
4	Merkel cell carcinoma	50 months (SFD 14; RFD 36)	PR	OFF therapy for 22 months due to irAE (Colitis)	Lymph nodes, retroperitoneum	No	RT to perinephric mass, pembrolizumab (Standard frequency)	PD	2 months	Died

*SFD* Standard frequency dosing, *RFD *Reduced frequency dosing, *BOR *Best overall response, *PR *Partial response, *SD *Stable disease, *PD *Progressive disease, *SRS *Stereotactic radiosurgery, *RT *Radiation therapy

Among the four patients with disease progression, three of the patients had previously discontinued systemic therapy at the time of progression. The time interval between cessation of immunotherapy and progression of disease ranged from 11 to 22 months. Two patients restarted immunotherapy, and one of the two patients experienced a partial response to anti-PD-1 therapy after reinitiating therapy

### Cost saving analysis

**Fig. 5:**
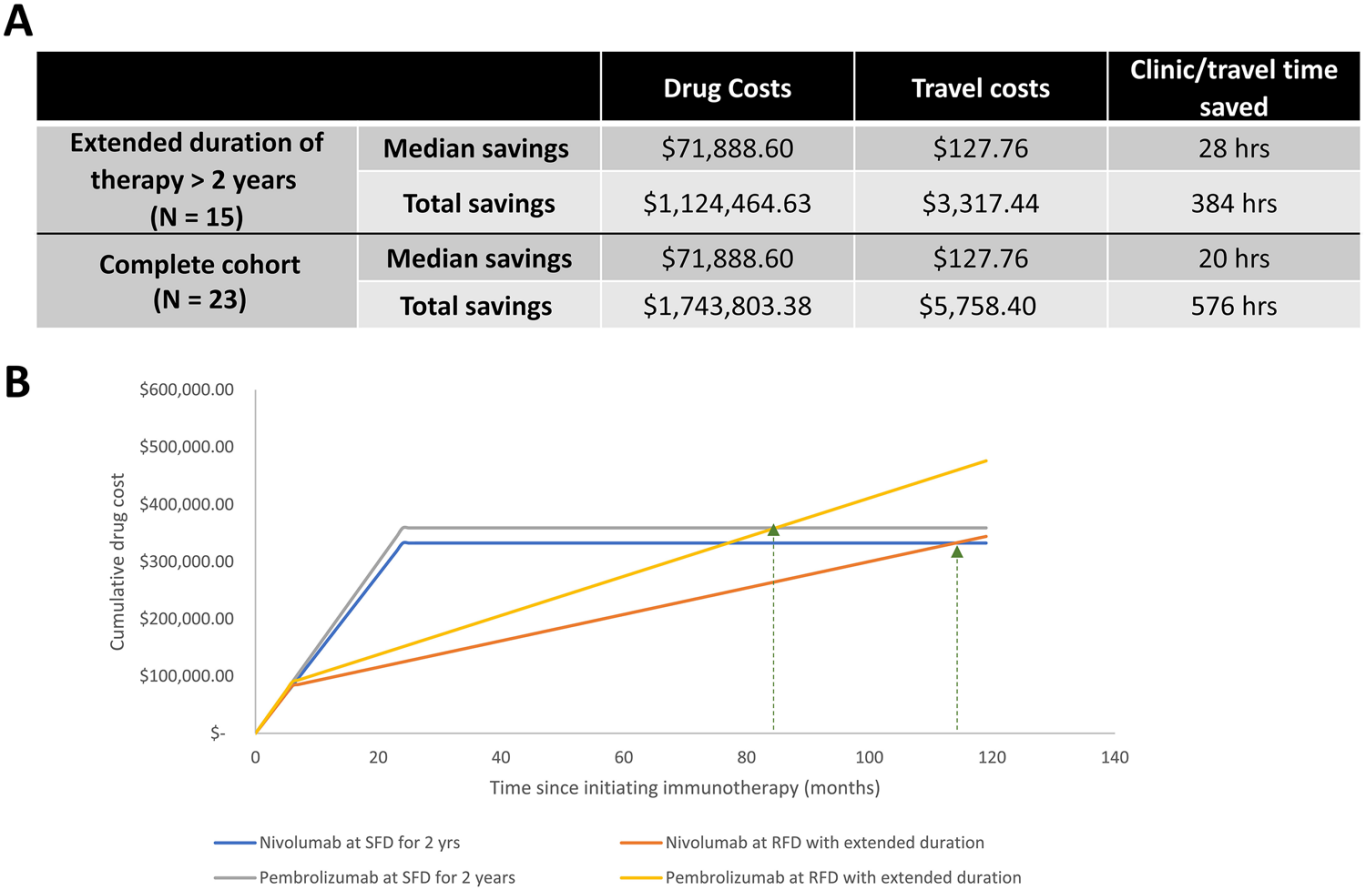
Savings with reduced-frequency dosing (RFD) **A** Among the subset of 15 patients with a total duration of therapy > 2 years (median 3.4 yr, range 2.0–5.0), we calculated savings from drug costs and patient-centered costs with the reduced-frequency approach (despite extended duration beyond 2 years) compared to the costs of 2 years of therapy at standard-frequency dosing (SFD). Additionally, the cost savings from drug costs and patient-centered costs were calculated for the entire cohort. **B** In a hypothetical model utilizing RFD after an initial 6 months of SFD, the total duration of treatment was extended to 84 months for pembrolizumab with reduced-frequency approach or 114 months with nivolumab with the reduced-frequency approach without incurring any additional costs. *Abbreviations* SFD—standard frequency dosing, RFD—Reduced frequency dosing

The cumulative costs of therapy (including treatment at SFD plus at RFD) in the subset of patients in the RFD cohort who had received extended duration of therapy (beyond 2 years) were compared to the costs of therapy at SFD for 2 years total. At the data cutoff date, 15 (65%) of our 23 patients had received a duration of anti-PD-1 therapy beyond 2 years (median 3.4 years, range 2.0–5.0). In this subset, the RFD approach was associated with total savings of $1,124464.63 in drug costs, $3317.44 in travel costs to patients, and 384 h of clinic and travel time compared to SFD for 2 years (Fig. 5A). Cost savings were also evaluated for the entire 23-patient cohort. In addition to the cost savings of the 15-patient subset mentioned above, costs savings of the entire cohort included patients who did not complete 2 years of ICI therapy by the cutoff date. In patients who received less than 2 years of therapy, the costs incurred on RFD were compared to the costs needed to maintain the equivalent duration of ICI treatment at SFD. The costs savings of RFD approach amounted to $1,743803.38 in drug costs, $5758.40 in travel costs, and 576 h of clinic and travel time. In a hypothetical treatment strategy that uses SFD for 6 months followed by subsequent treatment at RFD (every 12 weeks), the total drug costs for 2 years of SFD therapy could extend the duration of total PD-1 blockade to 7 years with pembrolizumab and 9.5 years with nivolumab with comparable drug cost expenditure. (Fig. 5B).

## Discussion

With a median follow up of 49.0 months, we report long-term safety and efficacy outcomes in a unique cohort of patients who received treatment with a reduced frequency dosing (RFD) approach after achieving disease control with standard frequency dosing (SFD) of ICI. In this cohort, implementation of RFD allowed a substantial extension of the duration of treatment beyond the usual arbitrary time point of 2 years employed in several pivotal clinical trials [1, 22]. Both efficacy and safety data suggest sustained immune activation with RFD administration of ICI. Importantly, cumulative drug costs and clinic time were significantly reduced with RFD. Our data suggest that the RFD strategy may provide an alternative approach to extend the duration of therapy, with substantially lower financial and logistical burden for patients as compared to the commonly used SFD approach for 2 years of treatment.

The rationale for RFD was modeled after the pharmacodynamic data from phase 1 studies of anti-PD-1 antibodies demonstrating persistent PD-1 receptor occupancy up to 100 days in vivo [14]. In silico modeling of nivolumab and pembrolizumab also support reduced-frequency doses administered every 2–3 months [23]. Several prospective, randomized clinical trials studying regimens like RFD are underway internationally based on the pharmacodynamic properties of anti-PD-1 antibodies [24–26]

The PFS rates at RFD in our cohort compare favorably to the historical outcomes with ICI at SFD, recognizing limitations of cross-trial comparisons. In our melanoma cohort, the PFS rates at 3 years after transitioning to RFD were 100% in patients with CR, 89% in PR, and 50% in SD. For reference, the 5-year PFS rates in the nivolumab monotherapy cohort of the CheckMate-067 trial, for those patients who did not progress during the first 12 months of treatment, were 88% in patients with CR, 63% in PR and 50% in SD [10]; in the ipilimumab plus nivolumab combination cohort, the 5-year PFS rates were 81% in patients with CR, 79% for PR and 11% for SD [10]. Similarly, in our MCC cohort, RFD outcomes appear to be maintained compared to historical cohorts. In our study’s small MCC cohort, the PFS rate at 36 months after transitioning to RFD was 100% in patients with CR and PR. For reference, the long-term follow-up of MCC patients treated with pembrolizumab on the KEYNOTE-017/CITN-09 trial reported that 73% of MCC patients with initial objective responses were progression-free at 36 months after treatment initiation [27].

Conversely, several retrospective analyses of real-world outcomes in patients who discontinue ICI have reported lower PFS rates compared to historical outcomes in patients with ICI continuation [8, 9, 28, 29]. For example, in a cohort of 324 melanoma patients from the Netherlands, the 24 month PFS after anti-PD-1 discontinuation was 64% in patients with CR, 53% in PR, and 31% in SD [9]. Heterogeneity in patient populations and shorter treatment durations likely contribute to lower PFS outcomes reported in real-world data [9]. Retrospective analyses have suggested higher risk of disease progression after ICI discontinuation in high-risk clinical factors, including patients who have PR or SD as BOR, ICI treatment < 6 months, younger age, higher LDH, and history of brain metastases [8, 9, 30]. Our cohort included many patients with unfavorable disease characteristics, including 60% of patients with brain or visceral metastases at the start of anti-PD-1 therapy and 74% of patients who did not achieve CR with SFD. These high-risk factors likely contributed to clinician decisions to continue immunotherapy using RFD in these patients.

Similarly, MCC patients may be at high risk of disease progression after treatment discontinuation. Multicenter retrospective analyses of advanced MCC patients in Germany (N = 20) and Australia (N = 40) who achieved disease control with immunotherapy report high rates of progression (35–60%) after treatment discontinuation [11, 31]. In the absence of protocolized conditions to electively discontinue therapy and lack of clear clinical factors to identify patients at high risk of progression after ICI cessation, treatment discontinuation must be considered cautiously [32].

As an alternative to treatment discontinuation, RFD may provide a sustainable option to extend treatment duration. Extending intervals between ICI doses has already been approved by the FDA for pembrolizumab doses of 400 mg every 6 weeks [17, 33]. In a retrospective, multicenter study of patients with advanced non-small cell lung cancer, patients treated with pembrolizumab 200 mg at extended intervals (defined as ≥ 2 cycles of pembrolizumab at intervals > 3 weeks + 3 days) had outcomes comparable to patients who received standard dosing [34]. Efficacy outcomes from these studies, in combination with our findings, underscore the need to study prospective trials using immune checkpoint inhibitors at less frequent doses.

Reduced frequency dosing of ICIs has significant cost-saving potential for this expensive class of drugs. As indications for ICI use have expanded across numerous cancer types, ICI utilization and spending have concomitantly escalated [35]. Specifically, between 2014 and 2019, Medicare expenditure for ICIs rapidly increased by 1916% from $285 million to $5.75 billion [35]. Beyond the US, global healthcare expenditure has also been affected by the increasing demand for ICI treatment. In 2022 alone, the global sales of pembrolizumab amounted to US$20.9 billion, and nivolumab global sales were US$8.2 billion [36, 37]. Looking forward, global costs of ICI drugs are projected to grow from US$54.8 billion in 2022 to US$185.4 billion in 2030 [15, 38]. Rising healthcare expenditures necessitate the development of sustainable and adaptive treatment strategies that reduce resource utilization without compromising patient care. RFD uses 15% of the standard nivolumab dosing (or 24% of the standard pembrolizumab dosing) needed to deliver one year of ICI therapy. Based upon 2022 global sales, RFD would have saved $7 billion in nivolumab costs, or $15.9 billion in pembrolizumab costs, over the past year [36, 37]. Furthermore, these costs prohibit most of the global cancer population, especially low- and middle-income countries, from accessing ICIs [15, 39, 40]. Decreasing the costs of ICI delivery may lower barriers for patients to receive care in underserved regions on a national and global scale.

Limitations of our study include retrospective analysis, small cohort size, cross trial comparisons and selection of patients who were already responding to ICI therapy at transition to RFD. RFD was not used during the induction phase of ICI therapy prior to patients’ objective responses. Therefore, our results may not fully support the upfront use of RFD prior to initial clinical response. Keeping in mind these important limitations, the learnings from our study warrant judicious application to other practice settings. Our data raise intriguing hypotheses for further investigation of the RFD approach in prospective clinical trials.

Many clinical trials have arbitrarily implemented ICI treatment discontinuation after 2 years of therapy at SFD. Our experience suggests that a transition to RFD may be safely implemented earlier in the treatment course, perhaps at the time of achieving CR or at an arbitrary timepoint of 6 months in patients without CR, given that most patients achieve their best responses early in the treatment course. The receptor occupancy data from prior studies and observations of irAEs with the RFD approach suggest that transitioning to RFD should not compromise the evolution of ongoing anti-tumor responses over time in these patients.

Importantly, the RFD approach, with its favorable financial and logistical footprint, will also allow investigation of questions related to the optimal duration of treatment. The significant logistical and financial burdens incurred from prolonged duration of immunotherapy at SFD would be unsustainable for patients and the healthcare system. Thus, the RFD approach may provide a feasible treatment scheme to evaluate the clinical benefit of prolonging duration of treatment. These questions are especially relevant in patients with disease characteristics that predict a higher risk of progression after treatment discontinuation, such as those with PR or SD, who may benefit from persistent engagement of the immune system. It is also worth highlighting that clinical response status alone may not accurately quantify level of residual disease, as patients with CR may still have detectable circulating tumor DNA (ctDNA) [41, 42]. In future trials, tests such as clinical biopsy or ctDNA could be used to identify patients with residual disease that may benefit extended duration of treatment with RFD. Correlative studies including receptor occupancy and pharmacokinetic data may further define the optimal dosing and frequency of RFD and are currently underway.

To our knowledge, this is the first study to report clinical outcomes with RFD administration of ICIs in patients with advanced melanoma and Merkel cell carcinoma. Sustained clinical benefit and occurrence of irAEs during RFD administration in our cohort suggest ongoing immune activation with the RFD approach. Our findings support further investigation of ICI dose optimization studies. With increasing utilization of ICI across multiple tumor types, an optimized RFD schedule has significant implications for sustainable healthcare delivery by permitting longer duration of therapy at a lower cost compared to similar durations at standard dosing. Furthermore, there is an urgent need to improve ICI access for low- and middle-income countries to this expensive class of potentially life-saving drugs. The RFD approach may help to lower barriers to ICI access, thereby impacting cancer outcomes on a global scale.

## Data Availability

Relevant data and analysis are published in this manuscript. Data used during this study are available from the corresponding author on reasonable request.
