# Correction to: Extended duration of treatment using reduced‑frequency dosing of anti‑PD‑1 therapy in patients with advanced melanoma and Merkel cell carcinoma

**DOI:** 10.1007/s00262-023-03575-4

**Published:** 2023-11-18

**Authors:** Lisa May Ling Tachiki, Daniel S. Hippe, Karly Williams Silva, Evan Thomas Hall, William McCamy, Dane Fritzsche, Andrea Perdue, Julia Majovski, Thomas Pulliam, Daniel A. Goldstein, Joshua Veatch, Joel Ho, Paul T. Nghiem, John A. Thompson, Shailender Bhatia

**Affiliations:** 1 Department of Medicine, Division of Medical Oncology, University of Washington, Seattle, WA USA; 2 Department of Medicine, Division of Dermatology, University of Washington, Seattle, WA USA; 3 Clinical Research Division, Fred Hutchinson Cancer Center, Seattle, WA USA; 4 Davidoff Cancer Center, Rabin Medical Center, Petah Tikva, Israel

**Correction to: Cancer Immunology, Immunotherapy (2023) 72:3839–3850** 10.1007/s00262-023-03539-8

The original version of this article unfortunately contained a mistake. The corrected detail is provided below:

In discussion section, 6th sentence of the 10th paragraph which previously read:

In future trials, tests such as clinical biopsy or ctDNA could be used to identify patients with residual disease that may benefit extended duration of treatment with RFD.

Should read:

In future trials, tests such as clinical biopsy or ctDNA could be used to identify patients with residual disease that may benefit from extended duration of treatment with RFD.

